# Epidural abscess formation with an atypical pathogen following epidural steroid injection: A case report

**DOI:** 10.1097/MD.0000000000030495

**Published:** 2022-09-09

**Authors:** Jae Young Lee, Jun Woo Kim, Yong Jae Na, Taikon Kim, Seung Hoon Han

**Affiliations:** a Department of Rehabilitation Medicine, Hanyang University College of Medicine, Seoul, Korea.

**Keywords:** *Enterococcus faecalis*, epidural abscess, epidural injection

## Abstract

Subcutaneous and epidural abscesses following epidural injection are a serious but rare complication. Epidural abscesses are typically caused by *Staphylococcus aureus* bacterial infection. In this case presented here, the causative bacterium was *Enterococcus faecalis*.

**Patient concerns:**

A 67-year-old woman having chronic lower back and right leg pain with past history of 20 years of rheumatoid arthritis, diabetes mellitus, and osteoporosis (T-score: −2.7) visited the outpatient pain clinic. Magnetic resonance imaging (MRI) revealed L4-5 right central disc extrusion with inferior migration. We performed a continuous epidural block for 7 days without complications. After 10 days, she presented with worsened low back pain, erythematous skin change on the lower back, chilling, and elevated serum acute phase reactants.

**Diagnosis:**

The diagnosis was subsequently confirmed by MRI suggesting subcutaneous and epidural abscess. Blood and pus cultures showed the growth of E. faecalis.

**Interventions:**

Pigtail catheter drainage was performed and intravenous antibiotics (ampicillin-sulbactam) targeting *E. faecalis* were applied for 3 weeks. Oral antibiotics (amoxicillin/potassium clavulanate) were applied for 6 weeks after discharge.

**Outcomes:**

At the 2-month follow-up, improvement in both the clinical condition and serum acute phase reactants levels were noted.

**Lessons:**

Epidural injection can lead to a subcutaneous abscess that is further extended into the epidural space. One of the key factors is the presence of comorbid conditions, including diabetes mellitus and prolonged steroid usage due to rheumatoid arthritis.

## 1. Introduction

Subcutaneous epidural abscesses are rare but serious complications after epidural injection.^[1]^ A few cases involving subcutaneous epidural abscesses have been reported previously in the literature.^[2,3]^ The spinal epidural space is a space between the dura mater and vertebra that contains fat tissue and venous plexus. This venous network has bidirectional flow that allows for the spread of infection from local or distant foci. Thus, pathophysiologically, epidural abscesses are known to occur via 3 mechanisms: bacterial infection from neighboring infected structure, hematogenous dissemination, and iatrogenic inoculation. However, no source can be identified in 30%–40% of cases. Based on the literature, *Staphylococcus aureus* is the most common source of epidural abscess.^[1,4]^

Although less common than *S. aureus*, *Streptococcus* species and Gram-negative rods are other sources of infection in association with spinal procedures, including during the placement of catheters for glucocorticoid injections or surgery.^[1,5]^ Cervical epidural abscess following *Escherichia coli* infection subsequent to urinary tract infection has been reported previously.^[6]^ Also, gas-containing lumbar spinal epidural abscess due to *Enterococcus faecalis* without any spinal procedure or intervention has been reported.^[7]^

In the case presented here, the causative bacterial species was *E. faecalis*. To the best of our knowledge, this is the first case of an epidural abscess caused by *E. faecalis* after epidural steroid injection and we report the case with a literature review.

## 2. Case description

**Figure 1.**
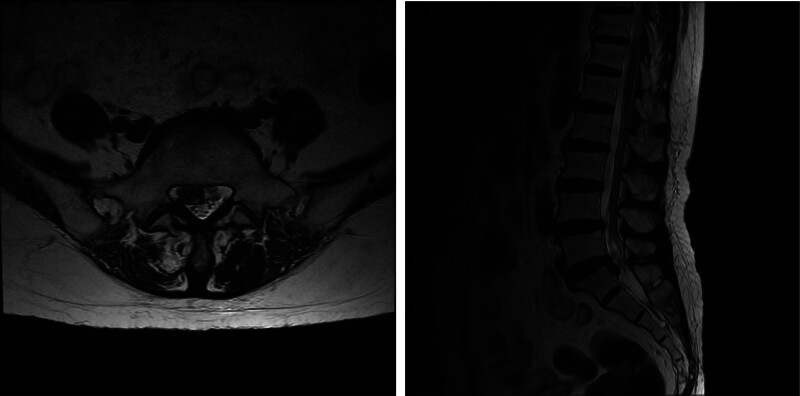
L-spine magnetic resonance images showing L4-L5 right central disc extrusion with inferior migration.

A 67-year-old woman having chronic low back and right leg pain with past history of 20 years of rheumatoid arthritis, diabetes mellitus, and osteoporosis (T-score = −2.7), had several trigger point injections at a local pain clinic. The pain was aggravated with persistent movement and radiated to the right hip and thigh. She had mild motor weakness in the right greater toe dorsiflexion and paresthesia on right L5 dermatome. Magnetic resonance imaging (MRI) revealed an L4-L5 right central disc extrusion with inferior migration (Fig. 1). Bilateral peroneal and tibial compound motor action potential (CMAP) and bilateral sural sensory nerve action potential (SNAP) and bilateral tibial F-waves were all within the normal limits. Electromyography (EMG) of bilateral lower extremities showed abnormal spontaneous activity and neuropathic motor unit action potentials in the right peroneus longus, tibialis anterior, and lumbar paraspinalis. These electrophysiologic findings were indicative of right L5 radiculopathy.

The patient had a medical history of diabetes and the use of associated medications for the previous 7 years. Her blood glucose levels had been well-controlled. We suspected that her symptoms were due to right L5 radiculopathy and performed a lumbar epidural block. After skin disinfection of the puncture area (L4–L5) using a solution of betadine (10% povidone-iodine) and ethanol, a lumbar epidural block was performed via a continuous technique with no difficulties.

**Figure 2.**
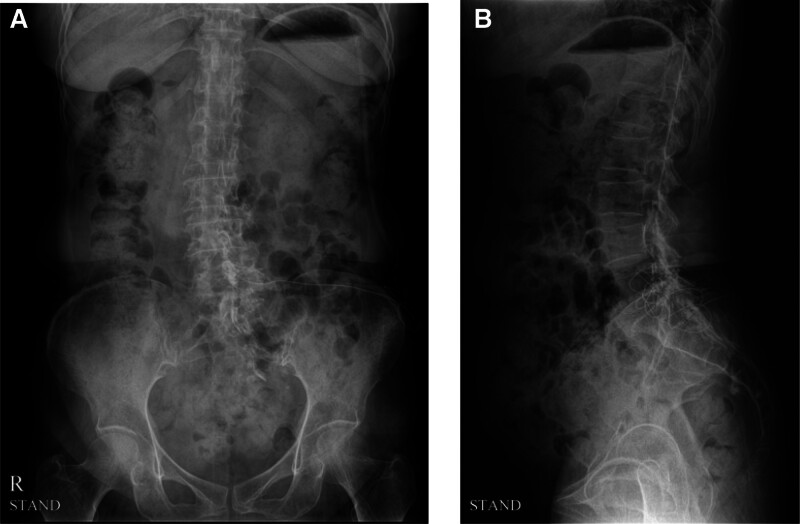
Lumbar spine radiography after contrast injection. The contrast showed that the catheter was placed in the epidural space in the (A) anteroposterior view and (B) lateral view.

An epidural catheter was introduced under aseptic conditions at a 90-degree angle. The bony structures were not grazed. Next, 5 mL 0.9% normal saline was injected to make sure that the catheter was in proper position. Lumbar spine radiography was performed after the injection of 8 mL contrast (4 ml 0.9% normal saline with 4 mL of contrast material), which showed that the catheter was placed in the epidural space (Fig. 2). The catheter was kept aseptically in place during the continuous epidural block, which was performed for 7 days. A mixture of 1 mL 2% lidocaine, 1 mL 0.5% bupivacaine, and 8 mL 0.9% normal saline was injected every day with additional triamcinolone on day one (triamcinolone 40 mg), day 4 (triamcinolone 20 mg), and day 7 (triamcinolone 40 mg). In addition, 10 mL 3% hypertonic saline was injected daily to induce adhesiolysis.

We checked vital signs 2 times a day for 7 days following the procedure. We also performed a daily physical examination for tenderness, skin color change, and heating sensation. Daily complete blood count (CBC) and C-reactive protein (CRP) tests were performed to monitor any signs of infection and no specific findings were identified. Oral preventive antibiotics were administered daily, and there was no evidence of infection, other events, or complications throughout the 7 days. The patient was discharged without any further complications, and her symptoms improved.

One week after discharge, she visited the outpatient department for a general checkup. Because her symptoms improved but continued, we performed a caudal epidural injection with 20 mg triamcinolone via a blind technique. A needle was inserted at 45 degrees into the sacral bone and after the needle was placed on the sacral hiatus, triamcinolone was injected.

**Figure 3.**
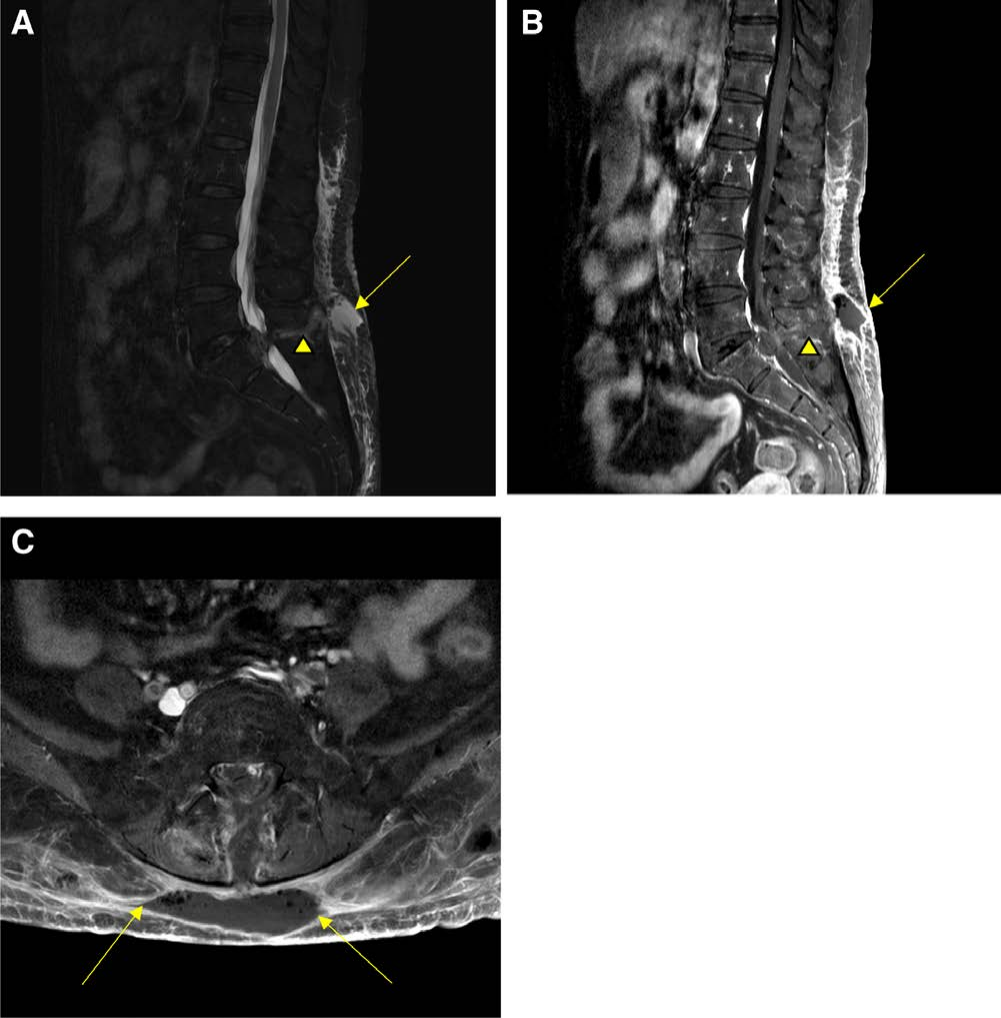
Lumbar magnetic resonance images suggesting a subcutaneous epidural abscess. (A) Subcutaneous abscess (arrow) and extension to the interspinous area at L4-5 (arrowhead) in a sagittal T2 weighted image. (B) Subcutaneous abscess (arrow) and extension to the interspinous area at L4-5 (arrowhead) in a contrast-enhanced T1 weighted image. (C) Gas-containing subcutaneous abscess (arrow) in an axial T2 weighted image.

**Figure 4.**
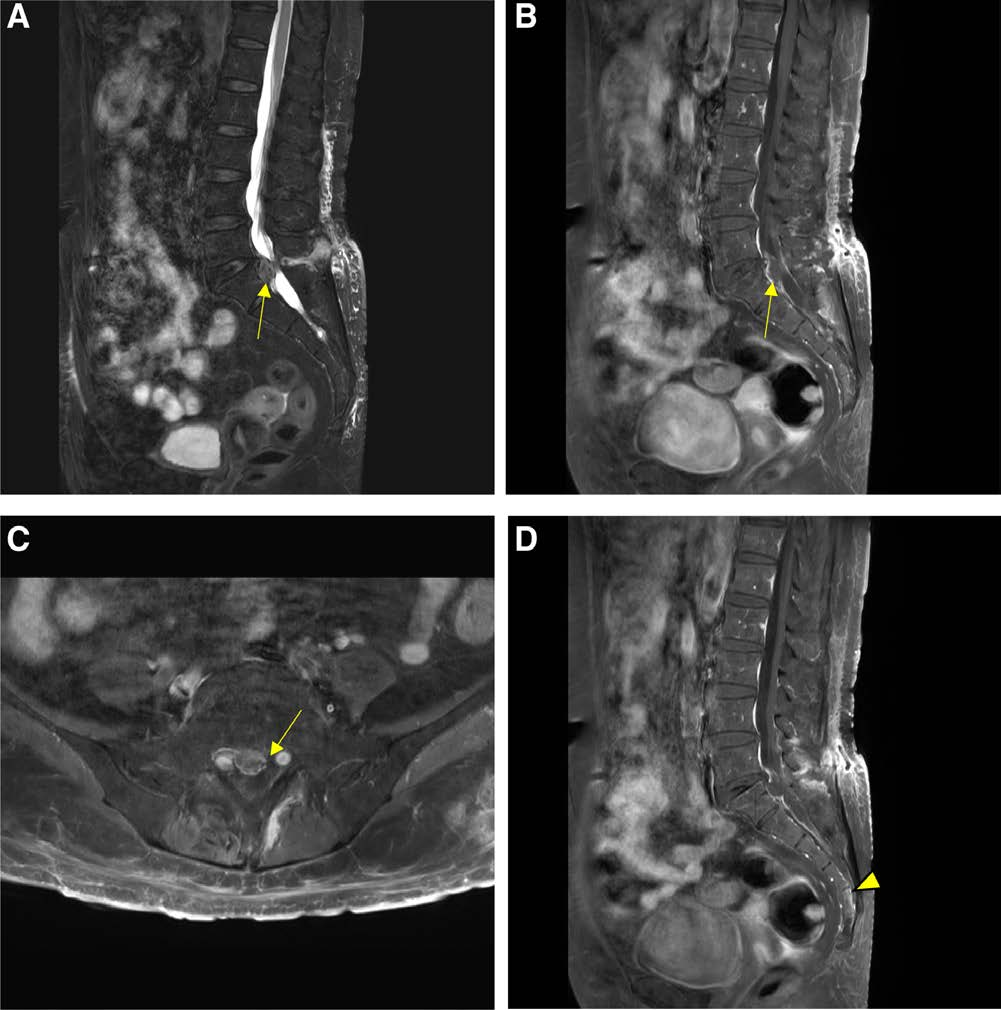
Follow-up lumbar magnetic resonance images after the drainage. (A) Abscess extended to epidural space of L4-5(arrow) in a sagittal T2 weighted image. (B) Abscess extended to epidural space of L4-5(arrow) with peripheral enhancing fluid collection in a contrast-enhanced T1 weighted image. (C) Abscess of epidural space of L4-5(arrow) with peripheral enhancing fluid collection in an axial contrast-enhanced T1 weighted image. (D) Bone marrow edema of S4-5(arrowhead) in a contrast-enhanced T1 weighted image.

Four days after the caudal block, she presented with worsened low back pain, erythematous skin change on the lower back, mild fever, and chilling. She was hospitalized and laboratory tests showed a white blood cell (WBC) of 9,300/mm^3^ and a CRP of 14.50 mg/dL. In assumption of bacterial infection, we performed blood and pus cultures via ultrasonography-guided subcutaneous aspiration. A subcutaneous abscess (size: 6.6 × 1.3 cm) was subsequently confirmed with MRI with extension to the interspinous area at L4-5 (Fig. 3). Pigtail catheter drainage was performed for the drainage of epidural abscess. After drainage, a follow-up MRI was performed, and the results suggested that the abscess extended into the epidural space of L4-5 and the spinal canal of the sacrum. In addition, bone marrow edema was identified in the sacrum, which seemed to be injured by the caudal epidural block (Fig. 4).

Pigtail catheter drainage was performed for the epidural abscess. Blood and pus cultures from the subcutaneous epidural abscess showed the growth of *Enterococcus faecalis* and intravenous antibiotics (ampicillin-sulbactam) targeting *E. faecalis* were administered. Because *E. faecalis* inhabits the gastrointestinal tract, and there was bone marrow edema in the sacrum, further examination was performed considering the possibility of colorectal puncture. Gadolinium lumbosacral MRI was performed to identify any fistula between the colorectal and epidural space; however, no fistula were identified. For identifying bowel perforation, abdominal computed tomography (CT) was performed, but no evidence of bowel perforation was found. Intravenous antibiotics were administered for 3 weeks during admission and oral antibiotics were administered for 6 weeks after discharge. At the 2-month follow-up, improvement in the clinical condition and serum acute phase reactant levels were noted.

## 3. Discussion

Here, we report the case of a patient with subcutaneous and epidural abscess following epidural injection. In general, *Staphylococcus aureus* is the most common cause of the epidural abscess.^[1]^ Nevertheless, in this case, the causative bacterium was *Enterococcus faecalis*.

*E. faecalis* is harmless as normal flora in the intestines; however, as an opportunistic pathogen, it can cause various infections including life-threatening bacteremia, endocarditis, meningitis, and urinary tract infection.^[8]^ Also, *E. faecalis* harbored on the skin is one of the most frequent opportunistic species identified in many wounds, including surgical sites, burns, and diabetic foot ulcers. *E. faecalis* can often spread through the hospital via healthcare workers and medical devices causing nosocomial infections. Immunocompromised patients are particularly susceptible to such infections.^[9]^

In this case, we considered 2 probable causes of *E. faecalis* infection. First, *E. faecalis* may have originated from the skin. By the same mechanism of wound infection, *E. faecalis* present on the skin could cause subcutaneous abscess and extend to the epidural space during the process of lumbar epidural injection.^[8,9]^ The patient had a history of rheumatoid arthritis and diabetes mellitus and had taken oral corticosteroid drugs for controlling rheumatoid arthritis. Because of these immunocompromised conditions, the patient may have been vulnerable to infection.

Second, colorectal puncture after the caudal epidural block may have caused the infection. The patient had a history of osteoporosis (T-score = -2.7) and there was bone marrow edema in the sacrum on MRI findings, which appear to be injured by the needle during the caudal block injection procedure. Considering the above findings, the needle could have injured the osteoporotic sacral bone and generated a puncture in the rectum anterior to the injured sacral bone. It is possible that the caudal epidural injection could have been contaminated by reintroducing the needle after puncturing the colorectal space, leading to a subcutaneous abscess, followed by further extension to the epidural space. However, subcutaneous epidural abscess due to colorectal perforation is less likely considering the gadolinium lumbosacral MRI and abdominal CT findings that showed no evidence of bowel perforation. In addition, considering the puncture site of the lumbar epidural and caudal epidural block, the location of the subcutaneous abscess was more consistent with that caused by the lumbar epidural block in this immunocompromised patient.

In this case, the treatment was successful with a protocol based on epidural abscess.^[1,10]^ The patient was treated with percutaneous catheter aspiration combined with antimicrobial therapy. Catheter aspiration is a diagnostic procedure that might also be therapeutic by reducing the size of the inflammatory mass. Only a few cases have been reported using this method and indication for this treatment is not well established. Patients with a posterior spinal epidural abscess without neurological deficit or with high surgical risk, and patients who do not fully respond to antimicrobial treatment, might benefit from this method.^[1,10]^

Epidural abscess is a serious condition that causes not only pain but also neurologic deficits and bacteremia.^[1]^ It can also provoke myelopathy compressing the spinal cord.^[11]^ Therefore, appropriate treatment of epidural abscesses is important. As mentioned previously, catheter aspiration combined with antimicrobial therapy is necessary. However, when physicians choose antibiotics for epidural abscesses, they have a tendency to focus on *S. aureus* because it is the most common species causing epidural abscesses. Considering this case, physicians should be aware of *E. faecalis* as a possible causative agent of epidural abscess following epidural injection, especially in immunosuppressed patients.

## 4. Conclusions

Epidural abscess following epidural steroid injection is a serious but rare complication. The case presented here is uncommon because the abscess formation was due to *E. faecalis*, and not due to typical *S. aureus*. Therefore, when physicians treat epidural abscesses following epidural injection, they should consider antibiotics targeting *E. faecalis* as well *as S. aureus*, particularly when treating immunocompromised patients.

## Author contributions

Conceptualization: SH Han.

Investigation: JY Lee, JW Kim, YJ Na.

Supervision: TK Kim.

Writing – original draft: JY Lee.

Writing – review & editing: SH Han, TK Kim.
